# The IRI-DICE hypothesis: ionizing radiation-induced DSBs may have a functional role for non-deterministic responses at low doses

**DOI:** 10.1007/s00411-020-00854-x

**Published:** 2020-06-24

**Authors:** Britta Langen, Khalil Helou, Eva Forssell-Aronsson

**Affiliations:** 1 Department of Radiation Physics, Institute of Clinical Sciences, Sahlgrenska Cancer Center, Sahlgrenska Academy, Sahlgrenska University Hospital, University of Gothenburg, SE-413 45 Gothenburg, Sweden; 2 Department of Oncology, Institute of Clinical Sciences, Sahlgrenska Cancer Center, Sahlgrenska Academy, Sahlgrenska University Hospital, University of Gothenburg, SE-413 45 Gothenburg, Sweden; 3 Department of Medical Physics and Biomedical Engineering, Sahlgrenska University Hospital, SE-413 45 Gothenburg, Sweden

**Keywords:** Low-dose response, DNA double-strand break, Cis effects, Transcription, Target theory

## Abstract

Low-dose ionizing radiation (IR) responses remain an unresolved issue in radiation biology and risk assessment. Accurate knowledge of low-dose responses is important for estimation of normal tissue risk in cancer radiotherapy or health risks from occupational or hazard exposure. Cellular responses to low-dose IR appear diverse and stochastic in nature and to date no model has been proposed to explain the underlying mechanisms. Here, we propose a hypothesis on IR-induced double-strand break (DSB)-induced cis effects (IRI-DICE) and introduce DNA sequence functionality as a submicron-scale target site with functional outcome on gene expression: DSB induction in a certain genetic target site such as promotor, regulatory element, or gene core would lead to changes in transcript expression, which may range from suppression to overexpression depending on which functional element was damaged. The DNA damage recognition and repair machinery depicts threshold behavior requiring a certain number of DSBs for induction. Stochastically distributed persistent disruption of gene expression may explain—in part—the diverse nature of low-dose responses until the repair machinery is initiated at increased absorbed dose. Radiation quality and complexity of DSB lesions are also discussed. Currently, there are no technologies available to irradiate specific genetic sites to test the IRI-DICE hypothesis directly. However, supportive evidence may be achieved by developing a computational model that combines radiation transport codes with a genomic DNA model that includes sequence functionality and transcription to simulate expression changes in an irradiated cell population. To the best of our knowledge, IRI-DICE is the first hypothesis that includes sequence functionality of different genetic elements in the radiation response and provides a model for the diversity of radiation responses in the (very) low dose regimen.

## Background

Radiation biology distinguishes between deterministic and stochastic responses, in principle depending on the absorbed dose level and biological endpoint under investigation (Little et al. 2009). The basic understanding of radiation responses is built on target theory that describes the relationship between absorbed dose and ionization events (‘hits’) in the DNA macromolecule. DNA is understood as the critical site (‘target’) for protracting damage that, ultimately, gives rise to health effects (Timofeeff-Ressovky et al. 1935; Elkind and Whitmore 1967; Alper 1979; Osborne et al. 2000). In this classical paradigm, DNA double-strand breaks (DSBs) are considered a severe (relevant) lesion, as opposed to single-strand breaks or other lesion types that are assumed to undergo (basically) error-free repair. In a simplified view, if the overall damage burden exceeds the capacity of the DNA repair machinery, cell cycle arrest is initiated to allow for extensive repair. Arrest and repair can result in fully recovered cells, recovered cells with silent or severe mutations, or cells undergoing apoptosis (Hall and Giaccia 2006). In the (very) low dose regimen, responses to ionizing radiation (IR) are more diverse and difficult to predict and, in particular, the relationship between absorbed dose, DNA damage, and health risk is an ongoing topic of debate. Low-dose radiation research is subject to the intrinsic dilemma that a low-dose stressor causes responses at low frequency and (generally) low intensity. This necessitates large data cohorts to demonstrate the causality between exposure and effect and to differentiate the effect (frequency) from the natural background frequency.

The linear no-threshold (LNT) model has been suggested for estimating stochastic effects in the (very) low dose regimen by extrapolation (National Research Council 2006; ICRP 2007; UNSCEAR 2008). Originally, the LNT model was developed for the purpose of radiation protection and only to be used when no data is available—meaning void of the claim to describe biological radiation responses. Nevertheless, mostly owed to the fact that epidemiological data on low-dose effects are scarce and composed of different endpoints and exposure conditions, there is an ongoing debate of the applicability of the LNT model for risk estimation (National Research Council 2006; Preston et al. 2013; Calabrese 2017; Mothersill and Seymour 2014; ICRP 2005; Tubiana 2005). This controversy is further fueled by the lack of omics data that could describe genome-wide responses to low-dose radiation beyond the focus on DNA damage and repair and related hallmarks.

In recent years, our group has contributed a host of transcriptomic data to the knowledge base on tissue responses to IR from i.v. administered radionuclides ^211^At (Rudqvist et al. 2012, 2015a, b; Langen et al. 2013, 2015a), ^131^I (Rudqvist et al. 2015b, c, 2017; Langen et al. 2015b), and ^177^Lu (Schüler et al. 2014a, b); these studies also included several low dose and low dose rate settings over 24 h. The dose–response relationship for total transcriptional regulation was non-linear and interestingly, for a number of (very) low dose settings, transcript suppression exceeded overexpression distinctly. Profiling of enriched biological processes showed that transcriptional responses were diverse and tissue-specific. The observed dominance of transcript suppression could not be explained by a uniform cellular response (such as cell cycle arrest and subsequent halting of cell physiological processes) in the light of the observed diversity of transcriptional responses. In general, this finding was in agreement with studies by Zhao et al. (2006), who demonstrated that IR-induced effects are not preprogrammed genetic responses but depend on the tissue-specific origin.

There is a need for a hypothesis on the observed response diversity in the (very) low dose regimen to advance the scientific process on the matter of low-dose risk assessment. Based on the discovery of how DSB induction affects transcript expression (Shanbhag et al. 2010; Shanbhag and Greenberg 2010), the threshold behavior of DNA damage repair (Shanbhag et al. 2010; Huen and Chen 2010; Ismail et al. 2005), and our findings on diversity of cellular responses at (very) low dose and low dose rate (Langen et al. 2013, 2015a, b; Rudqvist et al. 2012, 2015a, b, c, 2017; Schüler et al. 2014a, b), we propose a mechanistic model that introduces DNA sequence functionality to target theory to describe the diversity of radiation responses in the (very) low dose regimen.

### Hypothesis to explain low-dose response diversity: IRI-DICE

In a pioneer study, Shanbhag and colleagues showed that DSB production reduced transcript levels using a specific damage induction and reporter construct: DSBs were induced in the vicinity of a specific promoter resulting in suppression of transcription of the promoter-controlled gene which was termed double-strand break-induced silencing in cis (DISC) (Shanbhag et al. 2010; Shanbhag and Greenberg 2010). Moreover, transcriptional arrest also occurs in actively transcribed genes (by RNA polymerase II (RNAPII)) if the DSB lesion lies in the gene core independent of promotor disruption (Pankotai et al. 2012; Kim et al. 2016; Iannelli et al. 2017). In this case, inhibition of elongation and reinitiation do not appear to be mediated by the DSB lesion itself, but through DNA protein kinase (DNAPK) activity involving the proteasome-dependent pathway (Pankotai et al. 2012; Iannelli et al. 2017). Furthermore, a repressive cis effect (from a DSB in a given gene) has also been demonstrated for closely proximal genes (up to 100 kilobases (kb) distance), while distal genes [one megabase (Mb) distance] were basically unaffected (Iannelli et al. 2017).

DSB lesions are a hallmark of IR-induced damage. It can be assumed that the effect resulting from a DSB lesion is independent of the cause of damage if the DSBs from different sources present the same features and lesion complexity. Based on the molecular mechanism DISC described by Shanbhag and colleagues (Shanbhag et al. 2010; Shanbhag and Greenberg 2010) and supported by other groups (Pankotai et al. 2012; Kim et al. 2016; Iannelli et al. 2017), we postulate that IR-induced DSB-induced cis effects (IRI-DICE) impair not only the structural integrity of the DNA, but also the functional integrity of the transcriptome and, ultimately, affect cellular processes. We hypothesize that IRI-DICE manifests particularly in the (very) low dose regimen since the DSB burden is below the induction threshold of the DNA damage recognition and repair machinery in contrast to higher absorbed dose (Shanbhag and Greenberg 2010; Huen and Chen 2010; Ismail et al. 2005). We reason that the stochastic distribution of IRI-DICE across the genome results in stochastic disruption of gene expression, which contributes to the diversity of radiation responses observed at (very) low doses. (It should be noted that track-structure and genome organization may strongly increase the probability of a lesion to occur in a certain location in relation to a prior lesion.)

**Fig. 1:**
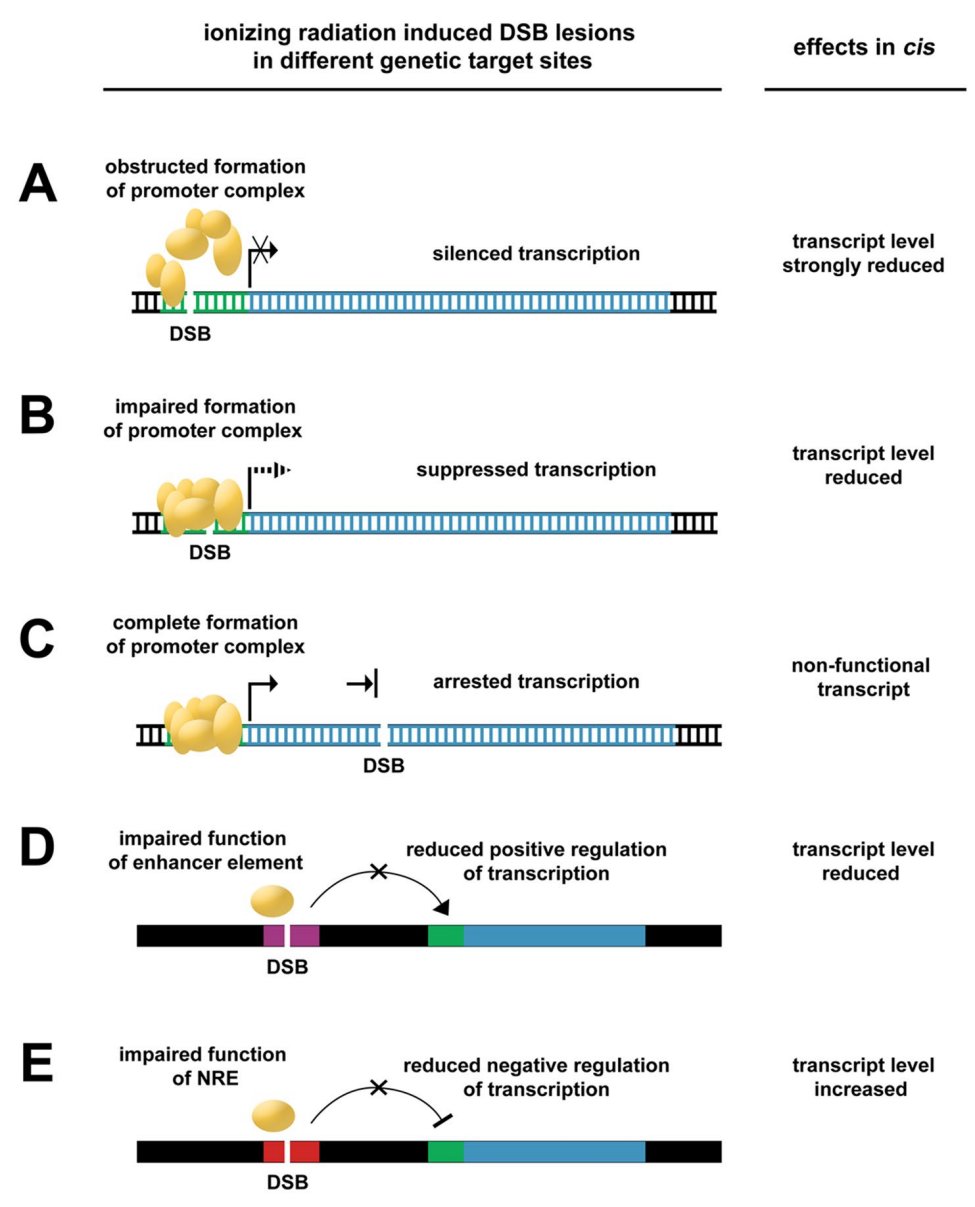
Illustrated IRI-DICE hypothesis showing the relation between DSB location and effect on transcript expression. The promoter region (green) is a DNA sequence where several proteins (orange) bind to form a promoter complex that regulates transcription (**a**–**c**). Inhibition of complex formation due to an ionizing radiation-induced DSB lesion results in strongly suppressed transcription (**a**), while improper binding due to DSB lesion can result in reduced transcription (**b**). In the gene core (blue), a DSB would not result in silencing of transcription, but in shortened and impaired messenger RNA (**c**). DSBs in regulatory elements can also affect transcript levels: a DSB in an enhancer sequence (purple) (**d**) or negative regulatory element (NRE, red) (**e**) can impair binding of transcription factors (orange) and have a negative or positive effect on transcription, respectively. Black arrows indicate activity or positive action; hashed arrow indicates reduced activity; bars indicate arrest or negative action; crosses indicate inhibition

Furthermore, the IRI-DICE model expands on target theory and introduces DNA sequence functionality as a genetic target site within the DNA macromolecule: the promoter region, regulatory elements, and gene core constitute genetic elements which, if damaged, would give rise to different functional outcomes. IRI-DICE could result in somewhat suppressed, strongly suppressed, completely suppressed (silenced), or even increased transcript expression, depending on which functional element was hit (Fig. 1). (Transcript increase, however, is considered less frequent due to the relatively short sequence length of negative regulatory elements (NREs) compared with the other genetic elements.) Accordingly, the outcome of IRI-DICE is thought to depend on the sequence length of different genomic target sites and the respective probability of being hit. Subsequently, if a gene product is not synthesized in the physiologically required concentration (at a given time) due to impaired transcription, the resulting cellular responses can be as manifold as the functions of genes expressed in a given cell type. In general, the impact of IRI-DICE on transcriptional integrity would also depend on whether the lesion occurred in an actively transcribed or inactive gene, i.e. the latter having no immediate cis effect. In this theoretic framework, epigenetic gene regulation (DNA compaction vs. accessibility) would constitute another factor that could influence the manifestation and frequency IRI-DICE and add to the complexity of low-dose radiation effects.

IRI-DICE may explain, in part, the stochastic nature of cellular responses observed in the (very) low dose regimen. Regarding increased absorbed dose levels, we reason that IRI-DICE would also occur initially, however, without lasting consequence: activation of Ataxia Telangiectasia Mutated (ATM) exhibits a threshold that correlates with the number of induced DSBs (Huen and Chen 2010; Ismail et al. 2005) and accordingly, initiation of (accurate) DNA repair would restore transcript expression. This reasoning is further supported by a study on DSB repair after X-ray irradiation, which demonstrated a higher frequency of persisting DSB lesions at a very low absorbed dose as opposed to an increased absorbed dose where less DSB lesions persisted (Rothkamm and Löbrich 2003).

## Discussion

### IRI-DICE and radiation quality

The frequency and persistence of IRI-DICE is expected to depend on radiation type, energy, and—notably, but not exclusively—the LET value. For a given absorbed dose, the ratio of hit vs. non-hit cell fractions and the probability of multiple hits within one cell differ between radiation types. For α-particle exposure in the micro- to milli-Gy range (mean absorbed dose), for instance, only a very small fraction of the cell population is hit by α-particles and would be subjected to (clustered) DNA damage. X-ray irradiation at the same absorbed dose would result in a higher fraction of hit cells and a higher probability of multiple (non-clustered) lesions within one cell, yet with a lower ratio of DSB compared with other lesion types. In context with the threshold behavior of the DNA damage recognition and repair machinery, the reconstitution of gene expression would be less likely for irradiation settings that lead to only a few DSB lesions per cell.

The fraction of protein-coding genes in mammalian genomes is relatively low with an estimate of less than 2% of genomic material (International Human Genome Sequencing Consortium 2001). Applying this gene-centric paradigm, a DSB in a relevant IRI-DICE target would only occur in approximately one out of 50 randomly distributed ionization events. This low ratio challenges the relevance that IRI-DICE would have for (very) low dose radiation responses if most DSBs were induced in sequences that have no function to be disrupted. However, various studies have demonstrated the correlation of long non-coding RNA (lncRNA) with human diseases, challenging the concept that vast regions of genomic material are without function or physiological relevance (Mattick 2004; Wilusz et al. 2009; Kung et al. 2013; Marchese et al. 2017). Moreover, the ENCODE (Encyclopedia of DNA Elements) project has supplied mounting evidence that over 80% of non-coding regions have at least one functional activity in one cell type (ENCODE Project Consortium 2012). It should be noted that ‘functional activity’ in this regard has been contested since the method detected primarily ‘chemical activity’ instead of endpoints with ‘biological function’ (Eddy 2012; Ford Doolittle 2013; Palazzo and Gregory 2014). Nevertheless, these regions have also been shown to underlie evolutionary selection (ENCODE Project Consortium 2012), which does support the claim of functional relevance even if the mechanisms have not yet been elucidated. Other work also indicates that a sharp distinction between coding and non-coding elements in terms of functionality may not be adequate when non-coding RNA can be derived from introns and exons of protein-coding and non-protein-coding genes (Mattick 2004). In addition, transcriptional ripple effects have been observed across the genomic landscape that affect expression kinetics in other regions beyond promotors and regulatory elements (Ebisuya 2008). Taken together with a suppressive cis effect on proximal genes (Iannelli et al. 2017), it is theoretically possible that a DSB induced in these non-coding regions may also constitute an IRI-DICE event with a cis effect on adjacent protein-coding genes. Alternatively, functional elements in the large non-coding regions of the genome (coding e.g., lncRNA) could be directly disrupted by a DSB similar to an IRI-DICE event (in a protein-coding gene). In either case, the probability of DSB-induced disruptive cis events greatly increases in the light of genomic sequence functionality beyond protein-coding genes.

Another important aspect regarding radiation quality is the complexity of DSB lesions. DSB damage can range from individual lesion sites to clustered damage (so-called locally multiply damaged sites, LMDS), which depends on the microdosimetric properties and can differ substantially between different radiation types and energies (Goodhead 1994; Ward 1994). It has been shown that damage complexity not only affects repair fidelity but also repair kinetics where more complex lesions persist longer than simple DSB sites (Pinto et al. 2005; Hable et al. 2012). Accordingly, not only the frequency of IRI-DICE would depend on radiation type and energy, but also the duration of transcript suppression, as clustered damage would persist longer than simple DSB lesion sites. For instance, complex DNA damage from α-particles would persist longer than the same lesion burden (i.e., number of DSB lesion sites) induced by X-ray irradiation. This would imply that radiation-induced DSB damage would not only have a functional effect on gene expression, but that physical radiation parameters would introduce a temporal dimension of how long that functional effect would persist.

### Approaches to test IRI-DICE

The IRI-DICE hypothesis offers a mechanistic model for the diversity and non-deterministic occurrence of cellular responses to (very) low absorbed dose. Technical advances in the quantification of DSB lesions have been made with e.g., BLESS, i-BLESS, BLISS, END-Seq or Break-Seq (Crosetto et al. 2013; Biernacka et al. 2018; Yan et al. 2017; Mirzazadeh et al. 2018; Canela et al. 2016; Hoffman et al. 2015). These methods allow for DSB detection with an improved spatial resolution that could be used to differentiate between lesions in the promotor region, regulatory elements, or the gene core. DSB location could then be correlated to single-cell transcriptional data of the same gene and thus support or reject the hypothesized IRI-DICE outcome (cf*.* Fig. 1). Unfortunately, while improved DSB detection is available, the localized irradiation of a specific genetic target site poses a limitation to experimental testing. So-called microbeam irradiations can be performed with sub-micron precision (Bigelow et al. 2009), but the beam diameter achieved to date is still too large to target a specific site in the DNA. Irradiation using radionuclides would theoretically be possible with specific DNA sequence-targeting molecules labeled with a radionuclide that only emits electrons with very low energy; however, to our knowledge, such compounds are not available at present. Hence, hypothesis testing with radionuclides or external beams is currently not available and would require major research and development efforts in the future.

A computational model may provide supportive evidence for the IRI-DICE hypothesis, but would necessitate a highly detailed chromatin model. The cell-type-specific model would need to be based on—most notably, but not exclusively—the nuclear distribution (i.e., size and localization) of protein-coding and non-coding genes, of actively transcribed or silenced genes, and of respective regulatory elements. The relative sequence length of functional sites would be an approximation of the probability to disrupt normal transcript expression of a given gene. Epigenetic regulation could be modeled as a binary multiplier (silencing or activation represented as zero or one). This genetic target site-specific model could then be used in Monte Carlo simulations of particle traversal and distribution of ionization events within a certain chromatin volume. To relate computational results to irradiation experiments, the simulation would also need to model tissues in vivo or cell cultures in vitro. Ultimately, this approach would yield the probability of DSB production in a certain gene and, according to the IRI-DICE hypothesis, the probability of observing a persistent increase or decrease in transcript expression. The in silico data should then be matched with experiments where a certain chromatin volume in the nucleus would be irradiated with a microbeam at (very) low absorbed dose. (While a genetic element cannot be targeted with current microbeam technology, it would limit the distribution of DSBs across the genome and thus reduce statistical complexity.) Immediately afterward, the cells would be subjected to single-cell transcriptional analysis and the data compared with unirradiated controls. This approach, however, bears critical limitations: for one, statistically large data cohorts would be needed from in vitro experiments, which poses a challenge to the throughput of microbeam irradiation and single-cell transcriptional analysis; for the other, genes function as a network, and transcriptional effects in one gene may cause a transcriptional response in another gene. Hence, data analysis (both in vitro and in silico) would need to consider transcriptional down-stream effects as confounders. It may thus be highly difficult to test the hypothesis until irradiation technologies are developed that allow DSB induction in specific genetic target sites.

## Concluding remarks

The IRI-DICE hypothesis provides a working model for the study of non-deterministic responses in the (very) low dose regimen by linking the stochastic occurrence of DSBs in a genetic target site to a functional effect on transcript expression. Furthermore, IRI-DICE suggests that DNA sequence functionality needs to be considered in an expansion of target theory for (very) low dose exposure and that functional elements are implemented in computational models in the future.

## Abbreviations

^131^I: Iodine-131
^177^Lu: Lutetium-177
^211^At: Astatine-211
ATM: Ataxia telangiectasia mutated (protein)
DISC: Double-strand break-induced silencing in cis
DNAPK: DNA protein kinase
DSB: DNA double-strand break
IR: Ionizing radiation
IRI-DICE: Ionizing radiation-induced DSB-induced cis effects
kb: Kilobase(s)
LET: Linear energy transfer
LMDS: Locally multiply damaged sites
lncRNA: Long non-coding RNA
LNT: Linear no-threshold (model)
Mb: Megabase(s)
NRE: Negative regulatory element
RNAPII: RNA polymerase II
